# Skin Grafting and Allied Procedures

**Published:** 1895-01-26

**Authors:** Alexander Miles

**Affiliations:** Surgical Tutor, Royal Infirmary, Edinburgh


### Medical Progress and Hospital Clinics.

[*The Editor will be glad to receive offers of co-operation and contributions from members of the profession. All letters should be addressed to* The Editor, The Lodge, Porchester Square, London, W.]

### SKIN GRAFTING AND ALLIED PROCEDURES.

By Alexander Miles, M.D., F.R.C.S. Ed., Surgical Tutor, Royal Infirmary, Edinburgh.

The various surgical procedures included under the generic term, "skin-grafting," differ from one another so widely as almost to involve a difference of principle as well as of means. They vary from the simple promiscuous scattering of epithelial cells over a granulating surface, to the transferring of an accurately measured and carefully prepared portion of skin to a corresponding raw area. It would appear desirable to apply to them names which would more accurately convey an idea of their nature. Some writers[1] have proposed to distinguish between *skin-grafting* and *skin-transportation*, applying the latter term to the operation of transferring the whole thickness of the skin, and the former to that of planting something less than the whole thickness. This distinction is neither anatomically nor philologically satisfactory, because in the so-called "skin-grafting" it is not strictly skin, but rather *epithelium* that is employed; and besides, while each is a "grafting" process, both are effected by "transportation."

If a quasi-horticultural nomenclature be adopted, it would seem that, without any attempt at a scientifically complete classification, the following might be a useful working arrangement of terms. (A) Epithelium sowing, M. Sée's method. (B) Epithelium grafting, (*a*) Reverdin's method; (*b*) Thiersch's method. (C) Skin-grafting or transplantation, (*a*) Human: (1) From patient himself; (2) from another person; (3) from amputated limbs; (*b*) from lower animals.

**Objects.**—All these means have as an end the rapid, healthy, and sound healing of a raw or granulating surface, in such a way as to produce a strong cicatrix, and one which will subsequently undergo a minimum amount of contraction. Of course the direction and degree of success varies with different methods.

**Suitable Conditions.**—The healing of any healthy open surface may be promoted by one or other of the above-named methods, but they are especially useful in cases of ulcers resulting from extensive burns; of large chronic ulcers of the lower limbs; in raw areas left after scraping lupus patches, excising epitheliomata, or removal of scirrhus of the breast; or in plastic operations for the cure of ectropion or maldeveloped bladder, &c. Opinions differ as to whether a fresh raw surface, a granulation-covered surface, or a granulating surface from which the superficial layer of granulations has been scraped, forms the most suitable soil on which to apply grafts. So far as the process is at present understood, the grafts live by becoming vascularised from below and so maintaining their vitality and powers of reproduction. The question therefore comes to be, from which of these surfaces young blood vessels will be most likely to speedily find their way into the applied graft? The recent raw surface has few small vessels on it, and these are running parallel with the surface of the skin, and are therefore little likely to enter readily the young graft. The vessels of a scraped granulation tissue have the same direction as the last, although they are much smaller and more numerous. The bleeding resulting from the scraping is a disadvantage, inasmuch as it prevents accurate apposition. The undisturbed granulating surface, on the other hand, consists in a large number of minute capillary vessels running at right angles to the surface of the skin, and in a state of active growth, with a very thin covering of embroyic cells, which the slightest pressure will displace, allowing the transported skin to come directly in contact with the vascular sprouts.

In practice, a healthy undisturbed granulating surface has been found to be the most generally satisfactory. Certain conditions of the ulcer are necessary to success. They are that the granulations be red and vascular, fairly equal in size, presenting a uniformly even surface almost flush with that of the surrounding skin. The discharge must be a small amount of healthy pus, absolutely sweet. The edges of the ulcer must be giving evidence of a tendency to heal in the form of a narrow, bluish ring of young epithelium spreading on to the surface of the granulations. There must be an absence of anything like œdema, or induration of the parts surrounding the ulcer, which, if they deviate at all from the normal, should be slightly hyperæmic, as indicating a vascular activity. These conditions can only be attained to if the ulcer be perfectly aseptic, and in this condition it must remain during the grafting process. At the same time, strong antiseptics are to be avoided, as they are apt to irritate and devitalise the young and delicate tissues. The grafts, of whichever variety, must be taken from a part which has been previously sterilised.

**Methods.**—(A) *Epithelium Sowing.*—This method, attributed to M. Sée, is so uncertain and haphazard as to be of little value. It consists in scraping the superficial cells of the epithelium with a knife edge, and scattering them indiscriminately over the surface of the ulcer. The scraping process, if it reach growing epithelium, is painful, and if it does not is useless, because only dead epidermis is removed. Usually but a scanty crop of epithelial shoots appear over the surface of the ulcer to aid healing. The result is infinitely less satisfactory than that obtained by Reverdin's method, of which it is an attempted simplification. It is not doing the thing well, and is therefore not worth doing at all.

(B) *Epithelium Grafting* (*a*) Reverdin's Method.—When Reverdin in 1869 suggested the transplanting of minute portions of vital epithelium and demonstrated the value of his method by a series of cases the procedure was recognised as a distinct and valuable advance in plastic surgery. Pollock[2], Bryant[3], Forster[4] and many others in this country speedily gave the simple method a trial with the most happy results, and the operation took a high place in surgical opinion. A portion of skin—preferably that of the patient himself, to avoid risk of transmitting disease—is purified, and a small fragment picked up on the point of a needle, and snipped off with sharp scissors curved on the flat, care being taken to cut through only the rete mucosum without drawing blood. This is then cut into pieces about the size of a pin's head, which are placed, deep surface down, at regular intervals over the surface of the ulcer, and gently but firmly pressed into the granulations. A dressing, consisting of protective, moist gauze, and wool, with gentle pressure from a bandage, is applied. On removing the dressings on the second or third day, the grafts may have entirely disappeared, but this does not necessarily mean failure, because they reappear in a day or two in the form of small bluish glazed areas of young epithelium, which rapidly increase in size and thickness. In their growth these seldom exceed in size a sixpenny piece. It is therefore advisable to plant the grafts not more than a quarter of an inch apart, and an equal distance from the margin, in order that they may fuse with one another the more rapidly and completely. Dressing every two or three days with warm boracic, or normal salt solution, avoiding all rubbing, and giving protection to the growing epithelium are indicated. (*b*) Thiersch's method: Another step forward was taken by Thiersch, who suggested the complete covering of the granulating surfaces by means of large thin sheets of epithelium. These he obtained by shaving off the more superficial layers of the skin, down to and including the mucous or Malphigian layer. A sharp hollow-ground razor is passed vertically through the skin till the Malphigian layer is reached and is then turned to lie flat, and with a rapid sawing movement a strip of epithelium about an inch to an inch and a half wide is shaved off and folds itself on the blade. Thiersch scraped away the superficial layer of granulations from the ulcer before applying the grafts; others to obviate the bleeding apply them on the surface of the granulations. In any case they should be laid close up to the margins of the ulcer and edge to edge with one another so as to completely cover the open surface. The free edge of the strip is fixed in position with the point of a needle, and by carefully withdrawing the razor the epithelium is acurately and evenly placed in position. Protective with moist gauze and wool form the dressing, and a firm splint will ensure immobility of the limb. The dressing may be changed every two or three days. The part from which the grafts are taken is dressed with a simple antiseptic dressing under which it heals.

(C) *Skin Grafting, or Transplantation of Skin.*—This method, of which the credit appears to be due to Dr. Wolff, of Glasgow, differs from those just considered in that the whole thickness of the skin is used. Inasmuch as the flap is entirely separated before being planted, it is by some spoken of as the British method, as distinguished from the Indian and Italian methods, in which a pedicle is left for the nourishment of the flap. The grafts, which should be from some soft, loose, and hairless region, may be taken from the patient himself, or from an accommodating healthy friend. Sometimes amputated limbs have been the source of the skin. A portion of skin, corresponding in size and shape to the area to be covered in, is dissected up from the subcutaneous tissue, completely removed, and at once transferred. In planning the operation an allowance of about one-third in every direction must be made for shrinking of the skin after cutting. It is not necessary to use stitches to retain the flap in position, as has been suggested by some. An ordinary aseptic absorbent dressing, with a layer of protective underneath, is sufficient for all purposes.

*Skin Grafting from the Lower Animals.*—The writer has employed the skin of such animals as dogs,[5] rabbits, kittens, and frogs in a series of cases, with variable but on the whole satisfactory results. The method differs but slightly from that of grafting from the human subject.

In all these procedures the key to success lies in having a healthy patient and sore, absolute asepsis, accurate apposition, and the most scrupulous care and patience in the after-dressings.

[1] Carmalt Buch's Handbk. Med. Sciences, Vol. II. [2] Pollock, Trans. Clin. Soc., 1871. [3] Bryant, Guy's Hosp. Rep., 1871-2. [4] Forster, ibid, 1872. [5] Miles, Lancet, March, 1890.

